# Characterization of Aniline Tetramer by MALDI TOF Mass Spectrometry upon Oxidative and Reductive Cycling

**DOI:** 10.3390/polym8110401

**Published:** 2016-11-15

**Authors:** Rebecca L. Li, Cheng-Wei Lin, Yuanlong Shao, Che Wei Chang, Fu-Kai Yao, Matthew D. Kowal, Haosen Wang, Michael T. Yeung, Shu-Chuan Huang, Richard B. Kaner

**Affiliations:** 1 Department of Chemistry and Biochemistry and California NanoSystems Institute, University of California, Los Angeles, CA 90095, USA; rebeccali@ucla.edu (R.L.L.); chengweilin@g.ucla.edu (C.-W.L.); mdkowal@gmail.com (M.D.K.); whs19890924@gmail.com (H.W.); tyrone@chem.ucla.edu (M.T.Y.); 2 Cambridge Graphene Centre, Cambridge University, Cambridge CB3 0FA, UK; ys461@cam.ac.uk; 3 Department of Chemistry, National Dong Hwa University, Hualien 97401, Taiwan; robin780115@hotmail.com (C.W.C.); sym23528@hotmail.com (F.-K.Y.); 4 Department of Materials Science and Engineering, University of California, Los Angeles, CA 90095, USA

**Keywords:** conducting polymers, oligomers, supercapacitors, degradation mechanism, mass spectroscopy

## Abstract

By combining electrochemical experiments with mass spectrometric analysis, it is found that using short chain oligomers to improve the cycling stability of conducting polymers in supercapacitors is still problematic. Cycling tests via cyclic voltammetry over a potential window of 0 to 1.0 V or 0 to 1.2 V in a two-electrode device configuration resulted in solid-state electropolymerization and chain scission. Electropolymerization of the aniline tetramer to generate long chain oligomers is shown to be possible despite the suggested decrease in reactivity and increase in intermediate stability with longer oligomers. Because aniline oligomers are more stable towards reductive cycling when compared to oxidative cycling, future conducting polymer/oligomer-based pseudocapacitors should consider using an asymmetric electrode configuration.

## 1. Introduction

In the search for materials for high-energy-density supercapacitors, much research has been carried out on conducting polymers, including polyaniline, polypyrrole and polythiophene [1,2,3,4,5]. When these materials are applied in pseudocapacitors, the bulk of the charge storage is derived from redox reactions and results in their high capacitance, energy density, and low cost. However, conducting polymers suffer from poor cycling stability. The generally acknowledged explanation is the degradation that results from the stretching and swelling of polymer chains [6,7]. To limit swelling and improve cycling life, composites of conducting polymers with carbonaceous material such as graphene, monolithic carbon, carbon nanotubes, and carbonaceous shells have been made [8,9,10,11]; this not only reinforces the mechanical stability, but also provides greater conductivity, surface area, and enhanced charge transport. Besides choosing materials to provide a good physical support for conducting polymers, the selection of a suitable operating potential window is arguably more crucial. A small potential window limits the charge storage ability, whereas a large potential window can cause over-oxidation and/or over-reduction of the conducting polymers, leading to device degradation.

Polyaniline stands out among conducting polymers for its ease of doping/dedoping and tunable conductivity. Upon partial oxidation from its leucoemeraldine base (LEB) form or partial reduction from the pernigraniline base (PNG) to the emeraldine base (EB) and doping with strong acids, conducting polyaniline salts are formed. The general rule of thumb when applying polyaniline to supercapacitors and improving cycling stability is to limit applied potentials below 0.6 V versus Ag/AgCl to avoid over-oxidation [12,13]. Studies carried out on the effect of potential on polyaniline have shown that over-oxidation occurs with the generation of degradation products such as quinoneimine species which accompany a great decrease in charge retention [12,14,15].

On the other hand, aniline tetramer is an appealing candidate with great potential for supercapacitors. With its shorter chain length, it is less susceptible to volumetric degradation that results from long chain swelling during the charging and discharging processes. As Yan et al. [13] have demonstrated, the aniline tetramer/graphene oxide composite exhibits good cycling stability after 2000 cycles using a potential window of −0.05 to 0.7 V versus Ag/AgCl. However, further reduction in the fluctuation of capacitance retention is only achieved with the narrowed potential window of 0 to 0.55 V versus Ag/AgCl [13]. The goal of this study is to use Matrix-Assisted Laser Desorption Ionization Time-of-Flight Mass Spectroscopy (MALDI TOF MS) to investigate any side products produced during cycling stability tests of aniline tetramer under different potential windows. Insights into the degradation mechanism of aniline tetramer can be gained and correlated with device operation which can greatly help in the design of future devices. In addition, a fundamental understanding of the response of aniline tetramer during oxidative or reductive cycling with varying potential can be achieved.

## 2. Materials and Methods

### 2.1. Synthesis of Graphene Oxide

In an ice bath, 20 g of graphite (ASBURY 3775, Warren, NJ, USA) was stirred for 20 min in pre-chilled concentrated sulfuric acid (Fisher, Waltham, MA, USA). KMnO_4_ (Fisher, Waltham, MA, USA) (120 g) was added over the course of 30 min to maintain the reaction temperature below 15 °C. After an additional 30 min of stirring the reaction mixture, ice was removed from the ice bath. The reaction was stirred for another 3 h in a water bath and quenched with 6 kg of crushed ice and 120 mL of 30% H_2_O_2_ (Fisher, Waltham, MA, USA). Synthesized graphene oxide crashed out after 24 h. Three washes with 10% HCl (Fisher, Waltham, MA, USA) and five washes with distilled water were carried out for initial purification. The graphene oxide was then further purified using dialysis for two weeks.

### 2.2. Synthesis of Aniline Tetramer [16]

First 0.8 g of *N*-phenyl-1,4-phenylenediamine (Sigma Aldrich, St. Louis, MO, USA) was dispersed in chilled 1.0 M HCl solution for 30 min. Then 1.4 g of ferric chloride (Merck, Elkton, VA, USA) was added in one addition and the solution was continuously stirred for an additional 2 h while ice was added to the ice bath to maintain the temperature at approximately 0 °C. The product was collected via centrifugation, de-doped with ammonium hydroxide, and continuously washed with a water and ethanol solution (1:3) for a minimum of 10 times. The purity of the product was confirmed via MALDI TOF MS.

### 2.3. Construction of Symmetric Devices

A solution of aniline tetramer was dispersed in ethanol and mixed with a solution of graphene oxide and carboxymethylcellulose/styrene-butadiene binder (MTI Corp., Richmond, CA, USA) through multiple sonication steps. The ratio of aniline tetramer to graphene oxide is maintained at a 5:1 ratio by weight. The ratio of total active material, aniline tetramer and graphene oxide to polymer binder is maintained at a 9:1 ratio by weight. The prepared solution was drop-cast onto 1.5 cm by 2 cm graphite foils with a controlled loading of 0.9 mg/cm^2^ and air dried. Copper tape served as the electrical connector and isolated from the electrolyte with Kapton tape. Supercapacitor devices were assembled with prepared slides, separator, 1.0 M H_2_SO_4_ electrolyte and secured with Kapton tape. All the cyclic voltammetry measurements were carried out using a Bio-Logic VMP3 potentiostat. Three-electrode measurements were carried out using platinum mesh as the counter electrode and a Ag/AgCl reference electrode.

### 2.4. Measurements

For zeta-potential measurements, the pH of the polyaniline or aniline tetramer solution was tuned by adding NaOH. Values of pH were determined using a pH meter (HANNA, HI9318, Woonsocket, RI, USA). The DTS cell was placed in a Malvern Zetasizer (Nano ZS 3600, Worcestershire, UK) for measuring the zeta potential.

**Figure 3:**
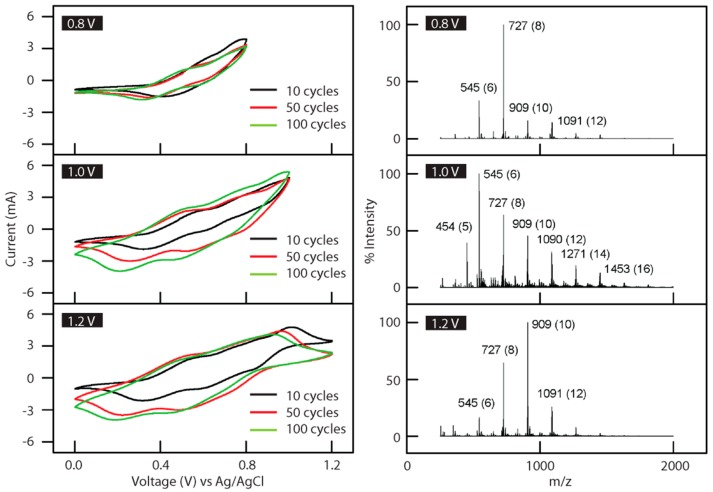
Electropolymerization of aniline tetramer in solution through repeated CV cycling with varying potential windows and the change in aniline oligomer distribution shown by Matrix-Assisted Laser Desorption Ionization Time-of-Flight (MALDI TOF) spectrometry.

Mass spectra for devices were collected by carefully scraping materials off current collectors. The analyte was dissolved in a mixture of ethanol and dimethyl sulfoxide (1:1) solution. A matrix solution was prepared which contained 10 mg of 2,5-dihydroxybenzoic acid (DHB) dissolved in 50 μL of acetonitrile and 50 μL of 10% trifluoroacetic acid (TFA) in water. Equal volumes of each solution were pipetted, mixed and dried on the MALDI TOF plate. Spectra shown in Figure 3 were acquired with an Applied Biosystems Voyager-De-STR MALDI TOF. Subsequent spectra were acquired with a Bruker UltraFlex MALDI TOF (Bruker Daltonics Inc., Billerica, MA, US). The UV–vis spectra were taken on a Shimadzu UV-3101 PC UV–vis-NIR Scanning Spectrophotometer with quartz cuvettes. The Scanning electron microscope (SEM) images were taken on an FEI Nova 230 (FEI, Hillsboro, OR, USA).

## 3. Results and Discussion

### 3.1. Polyaniline and Aniline Tetramer

**Figure 1:**
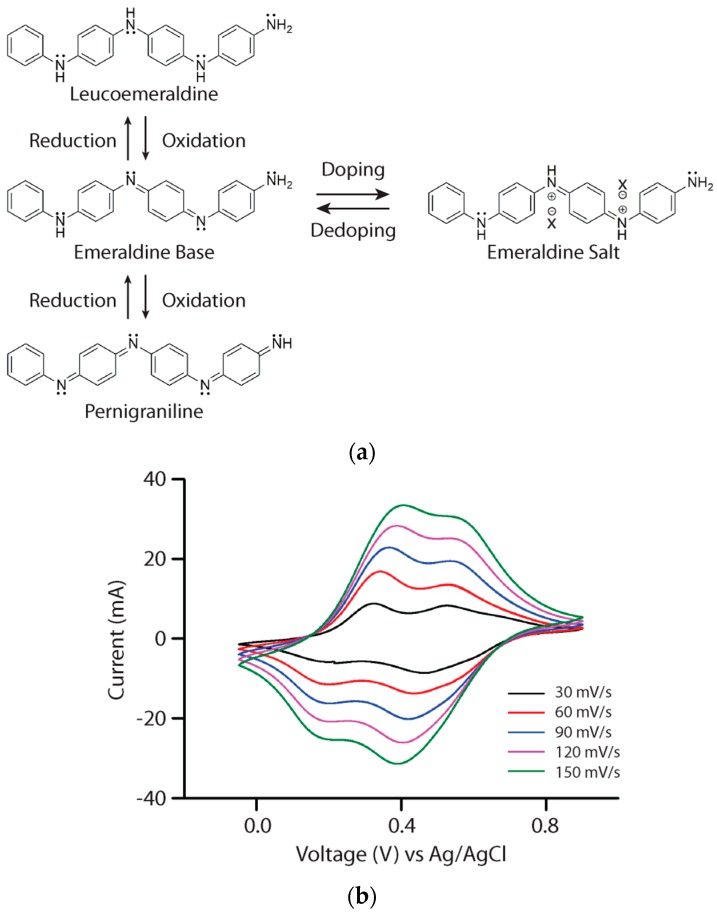
(**a**) Structures exhibiting the redox chemistry and doping properties of aniline tetramer; (**b**) cyclic voltammetry (CV) measurements on aniline tetramer film in a three-electrode configuration as a function of scan rate.

**Figure 2:**
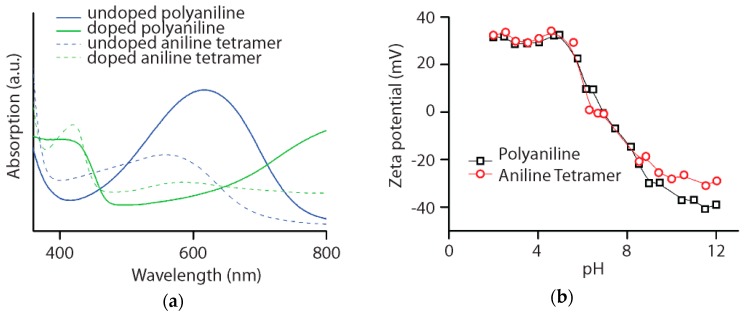
(**a**) UV–visible spectra of polyaniline and aniline tetramer demonstrating a color change from blue to green upon doping each in its emeraldine base state; (**b**) Zeta potential spectra of polyaniline and aniline tetramer exhibiting similar characteristics as a function of pH.

Aniline tetramer, the smallest representative unit of polyaniline [17], exhibits very similar redox and doping chemistry to polyaniline. Figure 1a illustrates the structures of the different oxidation states of aniline tetramer and the corresponding redox transitions. This is also illustrated through the electrochemical characterization of aniline tetramer in the solid state. Figure 1b shows the cyclic voltammetry (CV) plots obtained with varying scan rates in a three-electrode configuration using a potential window of −0.05 to 0.9 V vs. Ag/AgCl. In each of the CV curves, two redox peaks are observed during scans which correspond to the transitions between the three possible oxidation states; thus it is comparable to polyaniline [18]. The insulating emeraldine base (EB) state of aniline tetramer can be doped with acids to achieve the electrically conductive state known as emeraldine salt (ES). UV–vis spectra show the same doping chemistry for both polyaniline (solid lines) and aniline tetramer (dotted lines) in *N*-methyl-2-pyrrolidone (Figure 2a). The absorption peaks at around 600 nm for undoped polyaniline and aniline tetramer are attributed to the benzenoid-to-quinoid transition [19,20,21]. The emerging peaks at around 400 nm after acid doping are the characteristic peaks for bipolarons [22,23]. In solution, both aniline tetramer and polyaniline exhibit high zeta potential, conferring stable charged species at low and high pH values, as shown in Figure 2b. With almost identical doping chemistry and properties as polyaniline, aniline tetramer could be simply considered as polyaniline without its polymer nature.

### 3.2. Cycled Redox of Aniline Tetramer in Solution

In the experiments described above, aniline tetramer and polyaniline were shown to exhibit similar properties and chemistries. While polymerization from aniline monomer is commonly used to synthesize polyaniline, to the best of our knowledge, mass spectrum analysis on aniline tetramer electropolymerization under different potentials has not yet been reported. To understand the dependence of electropolymerization on the applied potential, repeated CV cycling tests were carried out on aniline tetramer dissolved in solution. This set-up allows the free movement of molecules and an increase in collision frequency. Experiments were carried out with 10 mM aniline tetramer with 1.0 M tetrabutylammonium perchlorate as the conducting electrolyte and 1.0 mM perchloric acid in acetonitrile. The addition of perchloric acid serves as a proton source so the deprotonation of aniline tetramer during oxidation can become reversible. A potentiodynamic technique was used where the applied potential is continuously varied in a cyclic manner within a potential window of 0 to 0.8 V, 0 to 1.0 V, and 0 to 1.2 V versus Ag/AgCl. These potentials were chosen so that aniline tetramer was charged with increasing levels beyond its different redox peaks in each experiment. Figure 3 shows the CV plots at cycles of 10, 50, and 100 to display the general trend during the course of electropolymerization. Note that the redox peaks observed in the spectra are less pronounced as aniline tetramer must diffuse to the working electrode for redox reactions to take place. In addition, studies done by Shacklette et al. have shown that through the use of crystalline active materials, sharper redox peaks were observed [24].

MALDI TOF data collected after 100 CV scans at scan rates of 5 mV/s with varying potential windows are also shown in Figure 3. MALDI TOF is an attractive technique due to the ease of spectral interpretation and peak assignments permitted by the soft ionization method which greatly reduces the fragmentation of the molecules [25]. Peaks corresponding to aniline oligomers are labeled as different n-mers in parenthesis along with the detected *m*/*z* values. It can be seen that the distribution of intermediate oligomer peaks shifts towards higher n-mers with increasing potential windows. In the potential window of 0 to 0.8 V, the aniline octamer (*m*/*z* = 727) has the highest percent intensity, whereas aniline 10-mer (*m*/*z* = 909) is the most dominant peak observed for the window from 0 to 1.2 V. With a potential window of 1.0 V, higher-order n-mers such as 14-mer and 16-mer were produced with low-percent intensity and lower-order n-mers were observed as the predominant species. For the distribution of peaks to shift to predominantly 10-mers, an even larger potential window of 1.2 V was required.

The trend in mass spectrum peaks supports the established notion that continuous polymerization of conjugated intermediates occurs only through increasing the reactivity of the system by applying a higher potential window. This is necessitated by the decrease in reactivity of longer-chain-length monomers. In addition, with increasing chain lengths, the acidity of the protons in charged and resonance-stabilized intermediates decreases, which hinders deprotonation and re-aromatization without sufficient energy in the system [26]. Closer examination of mass spectrum peaks shows the formation of oligomers with predominantly even numbers of aniline units. These data suggest that the chain breakage of oligomers must have occurred during the process of repeated CV cycling tests. If only the direct coupling of molecules had occurred, higher-order aniline oligomer peaks should only consists of aniline octamer, 12-mer and 16-mer. Instead, the oligomer peaks consistently differ by two aniline units. Note that chain breakage must also have occurred down to one aniline unit during polymerization to explain the appearance of the 5-mer in the 0 to 1.0 V potential window mass spectrum.

As shown above, the aniline tetramer polymerizes to a distribution of higher-order oligomer species when a potential is applied in a cyclic manner between 0 and the end-switching potentials specified above (0.8, 1.0 and 1.2 V). In each CV scan, the species in solution were oxidized and reduced through all three of the redox states. In order for aniline tetramer to be used in a supercapacitor application, the stability of the material as a function of the potential must be optimized. Therefore, symmetric supercapacitor devices were fabricated with aniline tetramer, predominantly in the EB state, and a graphene oxide composite to understand the experimental parameters of the potential and solid-state setup on aniline tetramer electropolymerization.

### 3.3. Aniline Tetramer as the Active Material in Supercapacitors

**Figure 4:**
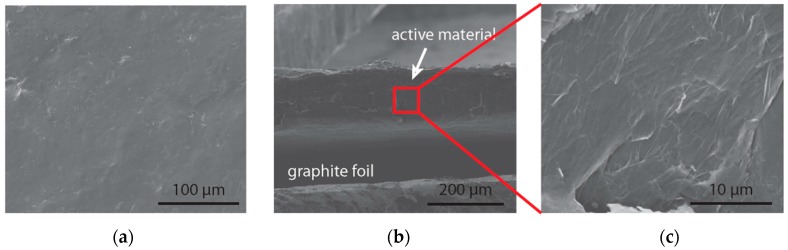
Scanning electron microscope (SEM) image of fabricated aniline tetramer and graphene oxide composite electrode: (**a**) top view; (**b**) cross-section of composite on current collector graphite foil; (**c**) expanded view of cross-section.

The active material containing aniline tetramer, graphene oxide flakes and polymer binders was deposited on graphite foil. Graphene oxide was added to provide aniline tetramer with a scaffold during repeated redox cycles, while the polymer binder was used to prevent the dissolution of the material. Graphene oxide was used instead of graphene in the fabrication of the electrodes because the relative hydrophilicity of graphene oxide allows for a more straightforward solution processing procedure and creates a more homogeneous solution of active material for drop-casting. The smooth and conductive film created can be seen in Figure 4a. A cross-sectional view of the electrode clearly shows the active material deposited on a graphite foil (Figure 4b). However, the expanded image does not show a typical layered graphene/graphene oxide structure [27] due to the relatively small amounts of graphene oxide added into the fabrication of the active material (Figure 4c).

**Figure 5:**
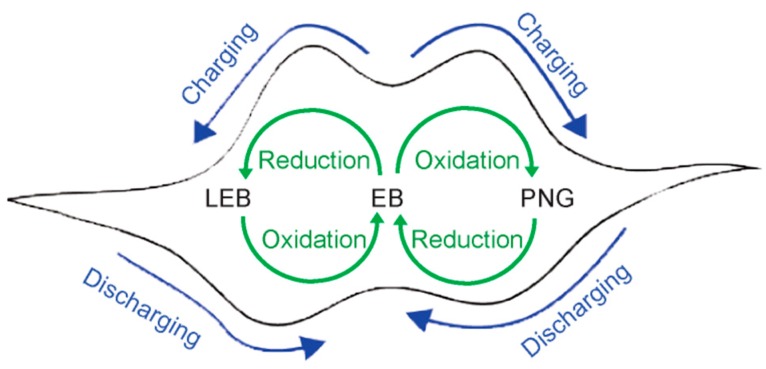
Schematic of the different redox transitions of aniline tetramer that occur on each electrode during device charging and discharging.

In a symmetric two-electrode setup, an electrodynamic equilibrium exists between the two identical electrodes, the applied potential should therefore be theoretically equally distributed over both electrodes. In a typical pseudocapacitor device setup, the material on the positive electrode is oxidized, while the negative electrode material is reduced during charging until the potential difference established between the two electrodes is equal to the set potential. Specifically in the fabricated devices of this study, transitions from the EB to the PNG (pernigraniline) state occur for one electrode during device charging, while the EB state is reduced to the LEB (leucoemeraldine) state for the other electrode. During device discharging, the PNG and LEB states are converted back to the EB state on the respective electrodes (Figure 5).

**Figure 6:**
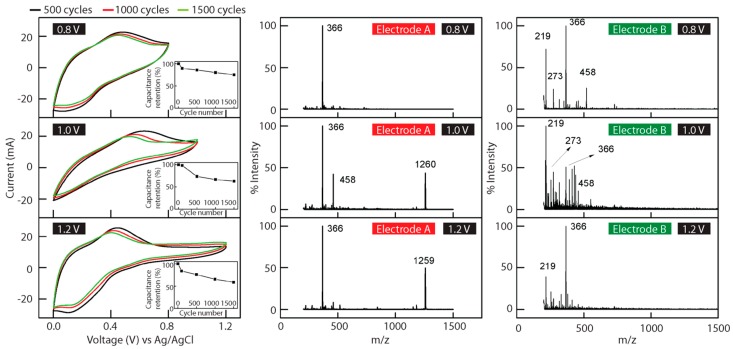
Select cyclic voltammetry spectra, taken throughout device cycling tests under different potential windows with their corresponding MALDI TOF spectra from electrodes A and B, showing peak polymerization and degradation after cycling. Inserts in the CV diagram plots show the percent capacitance retention as a function of cycle number.

The potential windows used for the devices were chosen to investigate the stability of the aniline tetramer with increasing levels of charging past the respective redox peaks. Figure 6 shows the CV graphs and MALDI TOF spectra for both sides of the electrode of three different devices with potential windows of 0 to 0.8 V, 0 to 1.0 V, and 0 to 1.2 V. The mass spectra peak with an *m*/*z* value of 366 corresponds to the EB state of the protonated aniline tetramer. For ease of spectra interpretation, only the CV spectra collected at 500, 1000, and 1500 cycles for each device with different potential windows are shown. In each set of CV curves, the redox peak observed demonstrates the Faradaic processes occurring in the system. The observed current plateau past the redox peak signifies a double-layer charge storage mechanism and is found in each device. There is a gradual shift in the anodic redox peak towards less positive potentials accompanying a decrease in the current intensity with the increasing cycle number. This suggests diminishing Faradaic charge/discharge properties with the degradation of some redox-active aniline tetrameric material. After 1500 cycles, the device with a potential window of 0.8 V retains 75.2% of its initial capacitance, the 1.0 V device maintains 62.9%, while only 58.6% remains for the 1.2 V device. In all the mass spectra (Figure 6), the aniline tetramer mass peak is still observed with a high percent intensity. This can be attributed to the dissolution of the electrode material during the course of electrochemical studies which is observed directly after disassembling the supercapacitor device for MALDI TOF testing. With the loss of charge and diffusion-controlled charge transport upon material dissolution, much of the aniline tetramer may have been prevented from undergoing redox reactions. The relatively rapid deterioration of the device performance can also be attributed to the dissolution of the active material.

The middle and right columns of Figure 6 show the MALDI TOF spectra of electrode side A and B upon 10,000 repeated CV cycles. A quick comparison between the mass spectra from both electrodes for the three devices shows an exceedingly different trend in the *m*/*z* values detected. Peaks with an *m*/*z* value as high as 1260 and 1259 are shown for electrode A of the devices with 1.0 V and 1.2 V potential windows, respectively. This peak can be assigned to the 14-mer aniline oligomer. In contrast, peaks signifying breakage of the aniline tetramer chains are observed for electrode side B. A 72.1% intensity peak with an *m*/*z* value of 219 is observed for the device with a 0.8 V potential window. A peak at *m*/*z* of 219 is observed in all spectra collected for electrode side B with varying intensities. Mass spectra corresponding to the electrodes for devices with potential windows of 1.0 and 1.2 V, respectively, demonstrate the effects of over-oxidation and over-reduction for aniline tetramer material. With repeated CV tests, the aniline tetramer undergoes degradation and solid-state polymerization on the different electrodes. In addition, the observance of higher-order aniline oligomer peaks for electrode A of 1.0 and 1.2 V as opposed to the 0.8 V device implies that similar to electropolymerization in solution, solid-state electropolymerization can only occur when sufficient energy is provided in the form of an increasing potential window. Likewise, shorter distances between chains could facilitate cross-linking or branching of aniline tetramer chains [28].

As demonstrated by mass spectrometry analysis, a major contributor to the poor cycling stability of the devices with large potential windows is the electropolymerization and degradation of the aniline tetramer material. However, the lack of polymerization for electrode side A and the significant degradation of material observed for electrode side B of the device with the potential window 0 to 0.8 V suggest an even more important general property for members belonging to the conducting polymer family. Specifically, the asymmetry in the capabilities of the aniline tetramer to undergo oxidative cycling and reductive cycling compromises the overall device cycling stability in a symmetric configuration. The relative instability of conducting polymers in the reduced state compared to their oxidized state has also been observed in studies carried out on other types of conducting polymers [29,30]. In the case of polythiophene, very negative potentials are required for reversible n-doping [2]. This potential is generally beyond the potential window range chosen for supercapacitor applications due to the limitation imposed by the stability of commonly used electrolytes [31]. Therefore, in symmetric devices, only reversible oxidative redox processes can take place. Furthermore, Borjas and Buttry have investigated the inconsistencies in the rigidity of polythiophene film during film growth under the application of negative potentials compared to positive potentials, which suggests instability of the film in a reduced state, and they propose the cause to be chain breakage [32]. In the case of the aniline tetramer, as Figure 6 demonstrates, there is a relative symmetry between the oxidation and reduction potentials which are both within the limits of commonly used electrolytes in the system. The mechanism by which aniline tetramer-based devices degrade is the generation of non-conducting species as shown by the MALDI TOF spectra. An additional factor compromising the overall device cycling is the lower stability of the negative electrode when undergoing Faradaic processes compared to the positive electrode, which causes both electrodes to be somewhat p-doped after being fully discharged [33]. Future pseudocapacitor device design using aniline tetramers should therefore choose a different electrode material with a stable negative potential window range and a n-doped cycling stability comparable to the p-doped cycling stability of the aniline tetramer.

## 4. Conclusions

In this study, the limitations of aniline tetramer in the field of supercapacitors are revealed which helps in deriving guidelines for future pseudocapacitor designs. Although it may appear that the mechanical instability of polyaniline can be ameliorated through using shorter-chain-length aniline tetramer, here, it is observed that aniline tetramer is susceptible to polymerization when a large potential window is applied. Electropolymerization of aniline tetramer dissolved in acetonitrile was also shown to be a viable method to form long chain aniline oligomers, generating large amounts of aniline 10-mer using a potential window of 0 to 1.2 V. The advantage of charging supercapacitor devices with higher potential windows is greater energy density. However, the caveat is the instability of materials past certain potential ranges which works against a long cycle life. MALDI TOF was used to monitor the change in the aniline tetramer after cycling under different potential windows. An asymmetry was observed in the stability between n-doping and p-doping cycling processes of aniline tetramer in a real-world two-electrode device configuration, which potentially contributes to the degradation of the overall device performance, even when using a narrow potential window of 0 to 0.8 V. Therefore, future pseudocapacitor construction designs with aniline tetramer should explore using asymmetric configurations where a different material that is not only stable in the potential range of the device, but also possesses n-doping cycling stability comparable to the p-doping process of aniline tetramer, is used.
